# Restricted Nitrogen and Water Applications in the Orchard Modify the Carbohydrate and Amino Acid Composition of Nonpareil and Carmel Almond Hulls

**DOI:** 10.3390/metabo11100674

**Published:** 2021-09-30

**Authors:** Anjali Zaveri, Jacqueline Edwards, Simone Rochfort

**Affiliations:** 1 School of Applied Systems Biology, La Trobe University, Bundoora, Melbourne, VIC 3083, Australia; Jacky.edwards@agriculture.vic.gov.au (J.E.); Simone.Rochfort@agriculture.vic.gov.au (S.R.); 2 Agriculture Research Victoria, 5 Ring Road, Bundoora, Melbourne, VIC 3083, Australia

**Keywords:** hull rot, *Rhizopus stolonifer*, fumaric acid, almond hull composition, irrigation, nitrogen treatment

## Abstract

Hull rot disease of almond (*Prunus dulcis*), caused by the fungus *Rhizopus stolonifer*, is prevalent in well maintained orchards where trees are provided plenty of water and nitrogen to increase the growth and yield. The predominantly grown variety Nonpareil is considered very susceptible to hull rot, while the pollinator variety Carmel is more resistant. Reduced nitrogen rates and restricted irrigation scheduling decreased the incidence and severity of hull rot in Californian orchards. As a part of our research, the hull composition of Australian almond fruits of Nonpareil and Carmel varieties, grown under two levels of irrigation (high and low) and two levels of nitrogen (high and low), were analysed using ^1^H NMR-based metabolomics. Both Nonpareil and Carmel hulls contained sugars such as glucose, sucrose, fructose and xylose, and amino acids, particularly asparagine. Variety was the major factor with Nonpareil hulls significantly higher in sugars and asparagine than Carmel. Within varieties, nitrogen influenced the relative concentrations of glucose, sucrose and asparagine. In Nonpareil, high nitrogen high water (the control) had relatively high glucose and asparagine content. High nitrogen low water increased the sucrose component, low nitrogen high water increased the glucose component and low nitrogen low water increased the sucrose and asparagine components. In Carmel, however, high nitrogen low water and low nitrogen high water increased sucrose and asparagine, and low nitrogen low water increased sucrose and glucose. Hull rot symptoms are caused by fumaric acid production by *R. stolonifer* growing within the hull. These changes in the hull composition under different nitrogen and water scenarios have the potential to affect the growth of *R. stolonifer* and its metabolite production in hull rot disease.

## 1. Introduction

Hull rot, a fungal disease of almond (*Prunus dulcis*), is considered the most serious disease of almond in Californian and Australian production systems [1,2]. It is of increasing concern to growers due to its prevalence in intensively managed and highly productive orchards [3]. Hull rot is mostly attributed to the fungus *Rhizopus stolonifer* and occasionally other *Rhizopus* and *Monilinia* species [4]. Recently, *Aspergillus niger* was also found to be associated with hull rot in California [5]. In Australia, hull rot has only been associated with *R. stolonifer* [6]. Fruit infected with *R. stolonifer* have dark grey to brown lesions along the opening of the suture line of maturing almond fruit with a visible dense, black fungal mass on the inner surface of the hull [4].

The fungus colonises the split hull and produces acidic metabolites [4,7] which are transported to the neighbouring shoots and leaves on the same vascular connection as the infected fruit. When the acids reach the spur, they cause leaf necrosis and spur death known as hull strike. Multiple occurrences of hull strikes on a tree result in reduced fruiting wood, reducing kernel production in subsequent years [1,4,7].

Hull rot is often worse in well-maintained orchards which are provided with plenty of nitrogen and water to increase growth and productivity [3]. There are several studies on the effect of varying nitrogen rates and scheduling and timing of irrigation on the incidence and severity of hull rot symptoms that support these observations. For example, restricting water supply to the almond trees as preharvest deficit irrigation significantly reduced hull rot in California [8,9,10]. Similarly, Teviotdale [11] investigated the effect of different nitrogen application rates on hull rot caused by both *R. stolonifer* and *Monilinia fructicola.* The number of hull rot strikes per tree and percentage of infected hulls were significantly positively correlated with the amount of nitrogen applied. She also observed that the rate of hull split was slower in the higher nitrogen treatments. Subsequently, Saa et al. [12] found that higher nitrogen increased hull rot by making hulls more susceptible to infection and was not related to the length of hull split time.

In a significant study in California, Mirocha and Wilson (1961) demonstrated the disease development cycle of hull rot [4]. According to their findings, *R. stolonifer* colonised inside the hull split but did not invade the hull tissue or colonise the affected leaf and twig tissue. They identified the involvement of toxin production by *R. stolonifer* that caused the leaf blighting and twig dieback [7] and identified the toxin using ^14^C-labelled mycelia of *R. stolonifer*. Their results pointed to fumaric acid as the candidate toxin with malic, citric and tartaric acid in the production pathway of fumaric acid. Zygomycetes, including *Rhizopus* spp., are well known for their ability to produce large amounts of primary metabolites in the presence of certain carbon and nitrogen sources [13,14]. *Rhizopus oryzae* produced 0.62 g lactic acid per g biomass when provided with glucose as the carbon source [15]. Other *Rhizopus* species have been investigated for the utilization of different carbon sources to produce fumaric acid, lactic acid and citric acid [16,17,18,19]. Due to this ability, *Rhizopus* species have been extensively studied for the commercial production of organic acids (i.e., fumaric acid and lactic acid), enzymes and ethanol [13,20,21,22,23,24]. This begs the question of whether restricting water and nitrogen to the almond tree modifies the carbon and nitrogen content of the almond hull, making it less favourable for acid production by *R. stolonifer* and limiting disease symptoms.

While there has been no research into hull composition with regard to hull rot disease, there has been considerable research investigating almond hull composition for use as an animal feed. Studies have shown that almond hulls consist of fermentable sugars fructose, glucose and sucrose, sugar alcohols sorbitol and inositol, polysaccharides and other components such as fibres, crude protein, soluble protein, minerals and nitrogen [25,26,27,28]. Variation in hull composition between different varieties have been reported [28,29], but to our knowledge, the relationship of in-orchard applications of nitrogen and water on hull composition has not been studied.

The current study utilised an NMR metabolomics approach to investigate the hull composition of two varieties of almond under four different nitrogen and water combinations applied in the orchards. NMR metabolomic approaches have been used previously to investigate factors affecting tomato composition [30]. The objectives of this study were to compare the influence of nitrogen and water on hull composition of two varieties of almonds, Nonpareil (hull rot susceptible) and Carmel (hull rot resistant), growing in an experimental site on a commercial orchard.

## 2. Results

**Figure 1:**
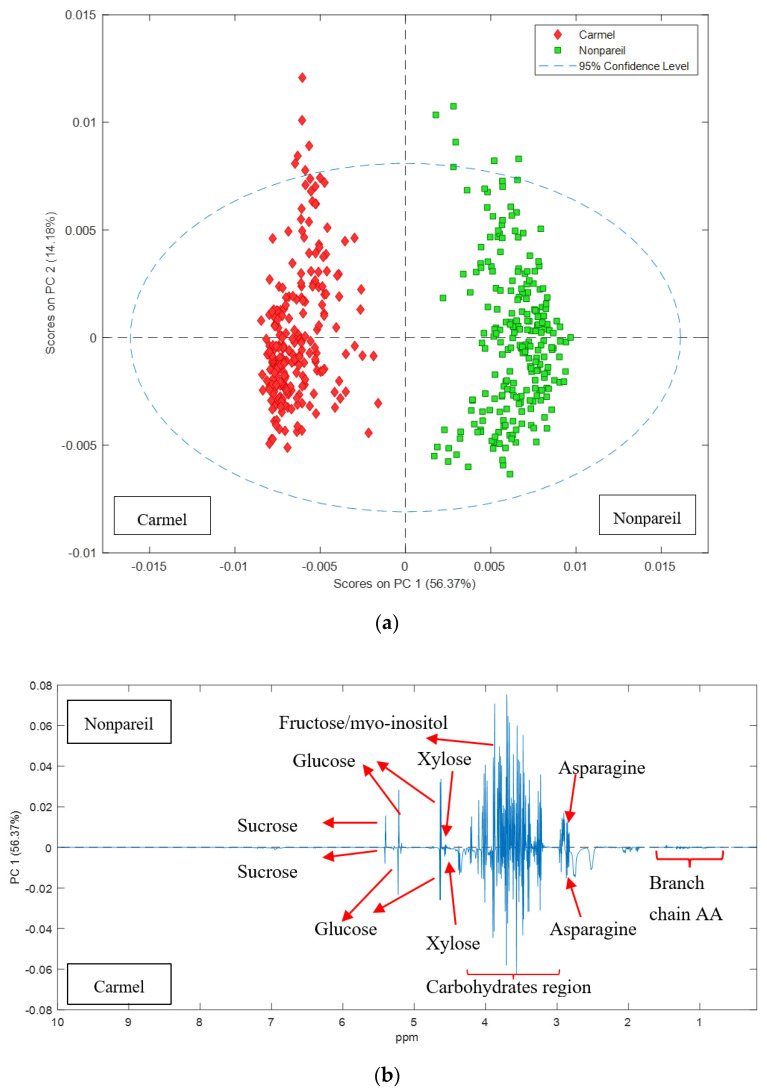
PCA score and loadings plots of 1H NMR data of Nonpareil and Carmel hulls: (**a**) PCA scores plot showing clear separation of Nonpareil and Carmel hull samples regardless of the treatment; (**b**) loadings plot of PC1 variables indicates that sucrose and asparagine are elevated in Nonpareil compared to Carmel.

Almond fruit (Nonpareil and Carmel) grown under two levels of irrigation (high and low) and two levels of nitrogen (high and low) were collected and the hull metabolites analysed by NMR. The NMR spectra showed that the extractable metabolome was high in carbohydrates and certain amino acids, in particular asparagine (Figure S1). In general, the hulls contained simple sugars, including glucose, fructose, sucrose and xylose, and the amino acids asparagine, alanine, valine, iso-leucine and leucine (Table S1). Principal Components Analysis (PCA) describes the variance between the samples and the largest observed difference in NMR spectra was associated with almond variety (Figure 1a), indicating that differences in hull composition due to variety were more significant than differences due to water or nitrogen applications in the orchard. Inspection of the loading plots revealed that this separation was due to higher sucrose and asparagine content in Nonpareil (Figure 1b). Given that almond variety was the predominant distinguishing variable, the NMR spectra for each variety were then analysed separately to determine the effects of the nitrogen and water applications.

### 2.1. Nonpareil Almond Variety

**Figure 2:**
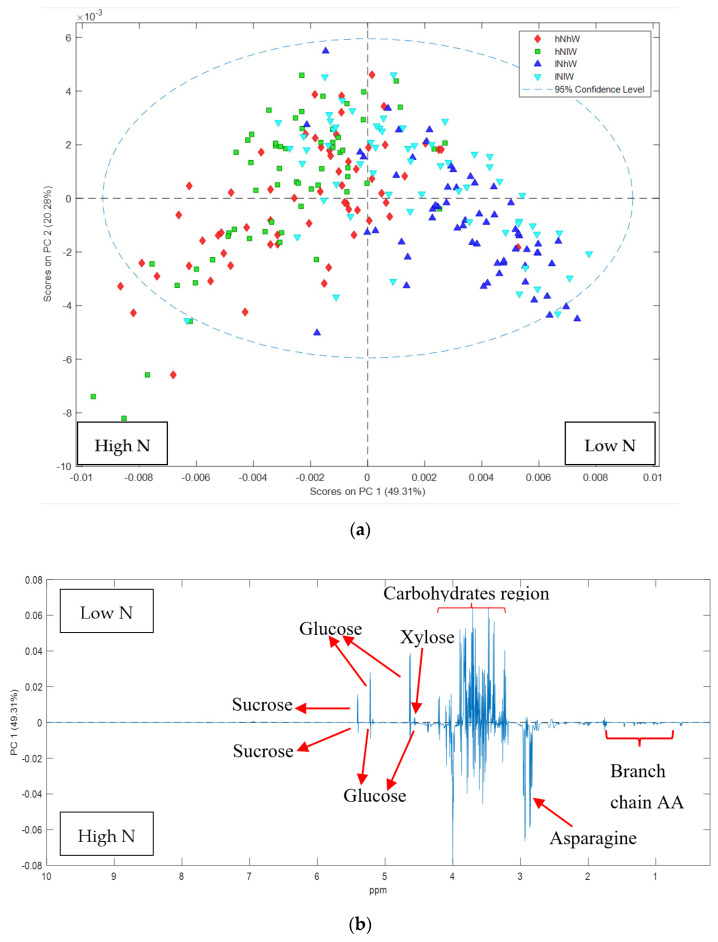

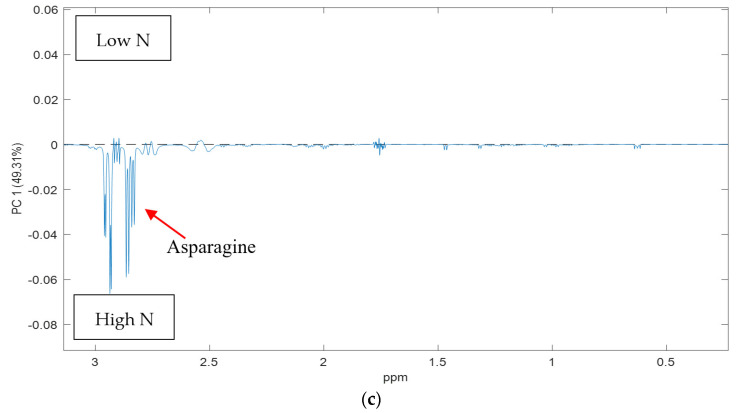
PCA score and loadings plots of ^1^H NMR data of Nonpareil hulls: (**a**) PCA scores plot showing clear separation of Nonpareil hull samples based on nitrogen treatment; (**b**) loadings plot of PC1 variables indicates that the high N treatment increased the amino acid content; (**c**) expansion of the loadings plot focusing on the amino acid regions of the spectrum.

The major separation in Nonpareil samples by PCA (Figure 2a and Figure S2a,b) was due to the different nitrogen treatments. The loadings plot showed that samples on the negative PC1 axis from the trees receiving high nitrogen (high N) had high levels of asparagine and other amino acids compared to those from low nitrogen (low N). Conversely, the high N spectra had relatively less glucose, sucrose and fructose which appear elevated under low N treatments (Figure 2b,c).

**Figure 3:**
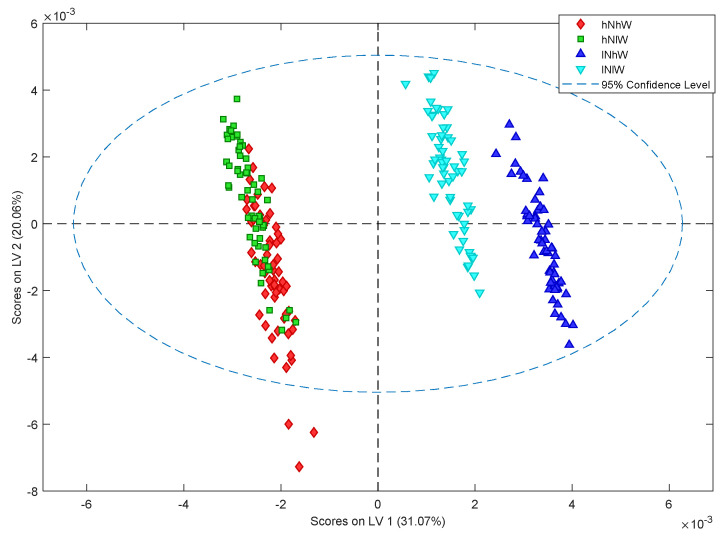
PLS-DA scores plot of ^1^H NMR data showing the major effect of nitrogen treatment on Nonpareil hull samples with significant water treatment influence on restricted nitrogen samples. Error of classification on cross validation: high N high W 17%, high N low W 21%, low N low W 11%, low N high W 19%.

**Figure 4:**
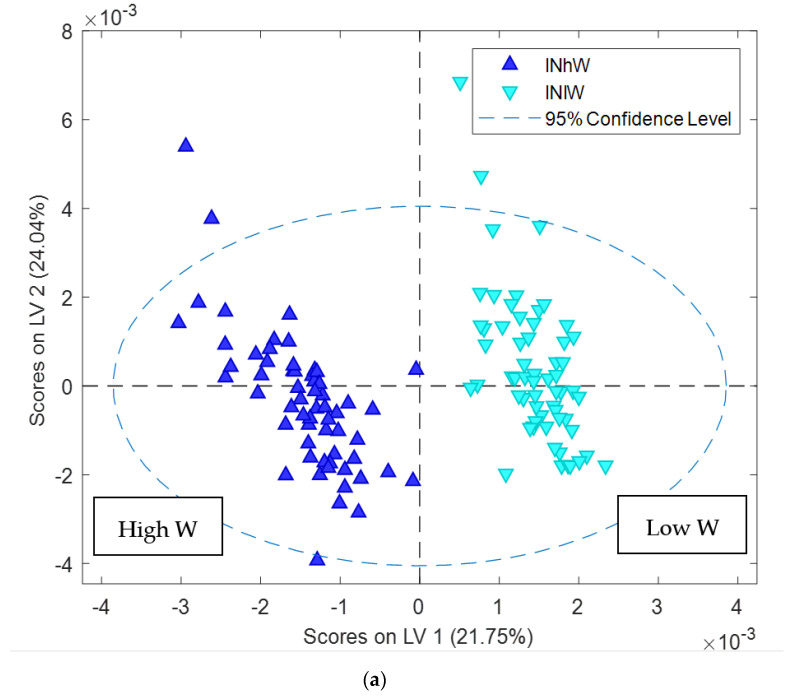

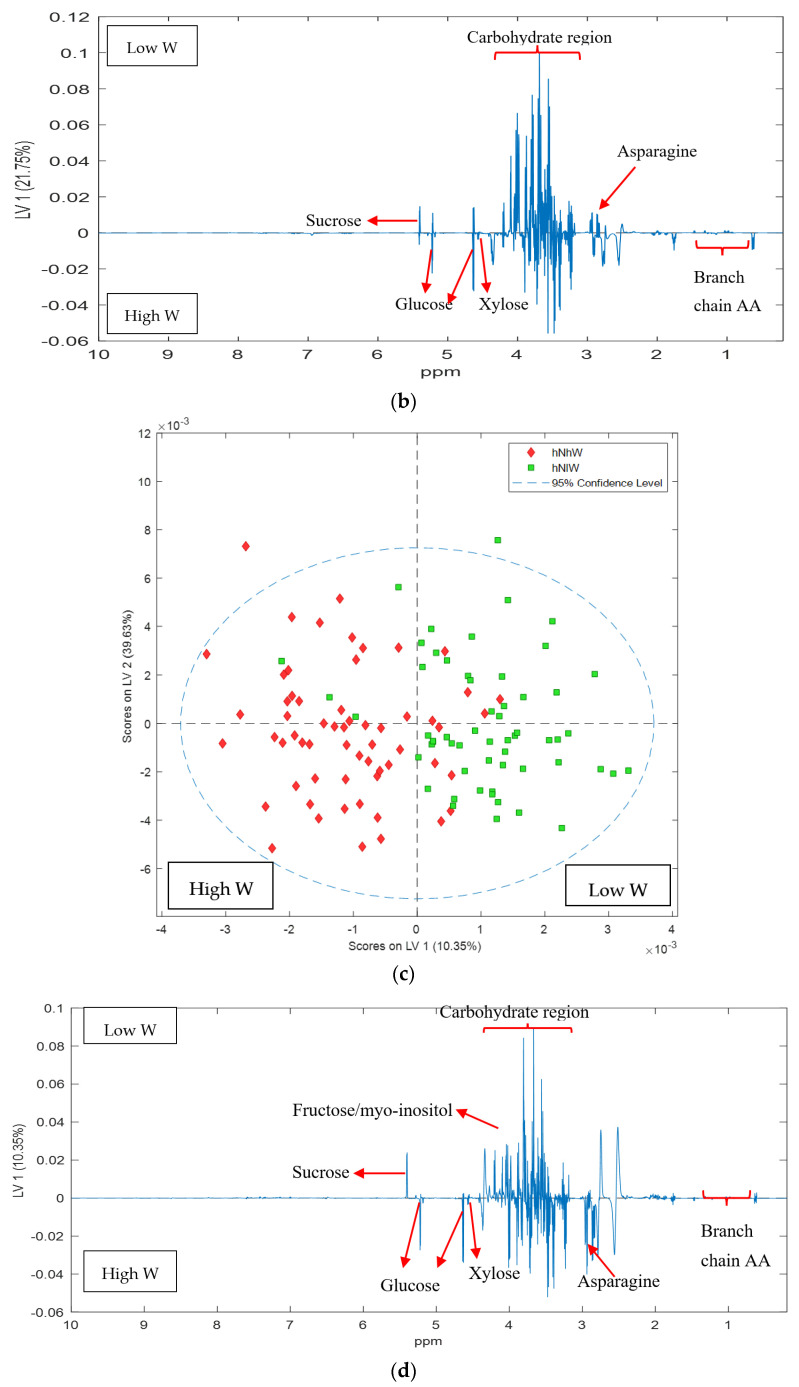
PLS-DA scores plot of ^1^H NMR data of Nonpareil hull samples: (**a**) PLSDA score plot showing effect of water treatments on hull composition of samples from low N treatment with classification error on cross validation of 4% and permutation *p*-value < 0.05 (**b**) Loadings plot of LV1 variable indicate the difference in hull composition components under water treatments of low N treatment (**c**) PLS-DA score plot showing effect of the high N treatments on hull composition with classification error on cross validation of 6% and permutation *p*-value < 0.05 (**d**) Loadings plot of LV1 variable indicate the differences in hull composition components under water treatments with high N treatment.

Classification modelling was conducted using a partial least squares-discrimination analysis (PLS-DA) to confirm the PCA results and to determine whether the effect of water treatments could be determined statistically. A PLS-DA model was constructed to classify samples based on the four treatment classes. The orthogonal signal correction (OSC) processed PLS-DA model was somewhat predictive for each class with classification errors between 11% and 21% on cross validation: high N high W 17%, high N low W 20%, low N low W 11%, low N high W 19%. The major discrimination in latent variable 1 (LV1) was due to differences in nitrogen treatment. However, particularly under low N conditions, a water effect could be seen (Figure 3). When each nitrogen treatment was analysed separately, clustering due to the water treatment was observed. The PLS-DA model of the low N spectra showed a strong influence of water treatment with classification error on cross validation of 4% and permutation *p*-value < 0.05 indicating the model significance at the 95% confidence level (Figure 4a). The PLS-DA loadings plot analysis showed an elevation of sucrose and asparagine under low N low W conditions (Figure 4b). The OSC processed PLS-DA model of high N samples also showed separation based on water treatment classification (Figure 4c). The classification error on cross validation samples was 6% with permutation *p*-value < 0.05. It was observed that under high N high W, asparagine and glucose were elevated (Figure 4d), while sucrose was elevated under high N low W.

### 2.2. Carmel Almond Variety

**Figure 5:**
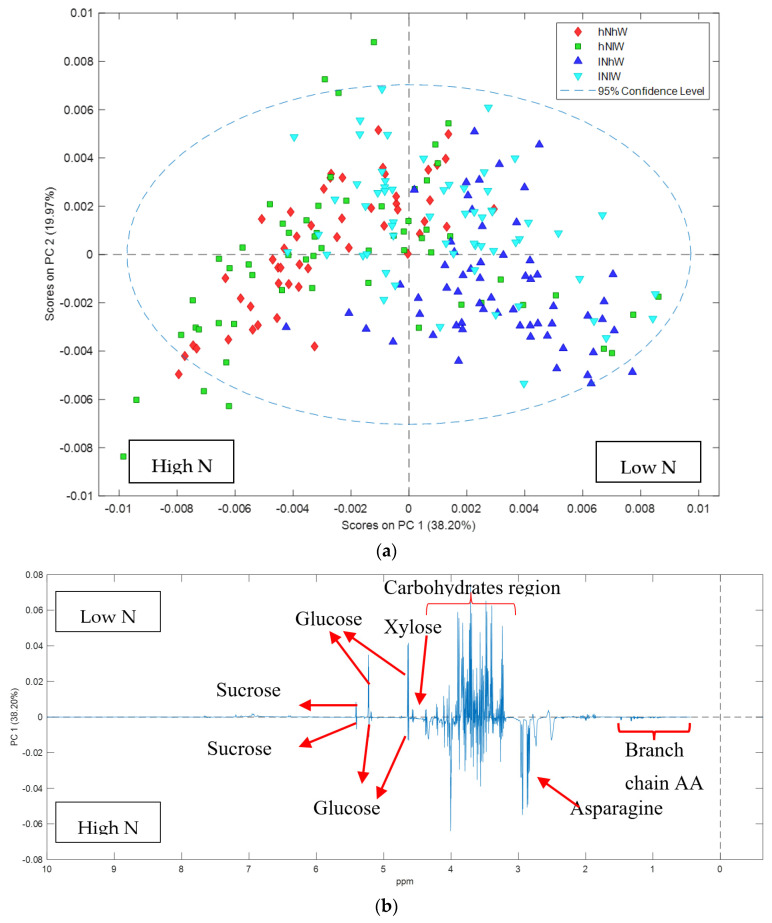
PCA score and loadings plots of ^1^H NMR data of Carmel hull: (**a**) PCA scores plot showing clear separation of Carmel hull samples based on nitrogen treatment; (**b**) loadings plot of PC1 variables indicates that the high N treatment increased the amino acid contents.

The Carmel hull spectra were analysed using PCA and PLS-DA, as for the Nonpareil hull samples. The major separation by PCA (Figure 5a and Figure S3a,b) was again due to differences in nitrogen treatments. The loadings plot shows that samples on the negative PC1 axis (high N) had higher levels of asparagine and other amino acids. Conversely, the high N samples had less glucose, sucrose and fructose than under the low N treatment (Figure 5b).

**Figure 6:**
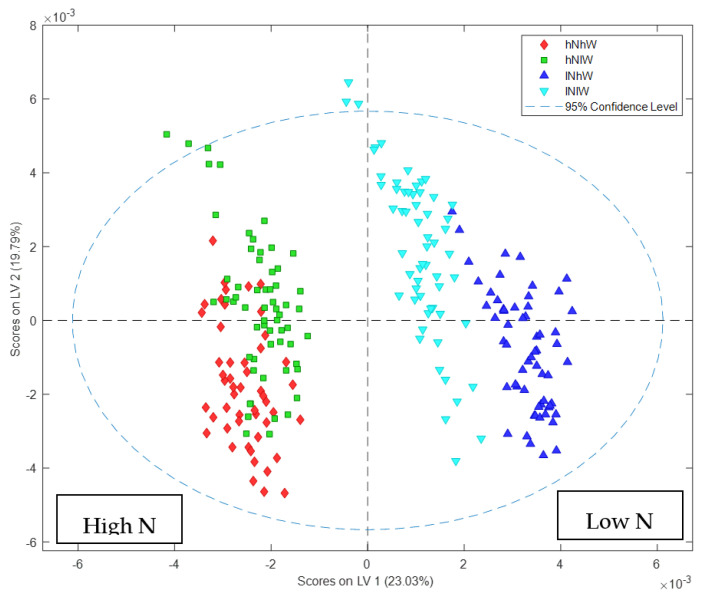
PLS-DA score plot of ^1^H NMR data showing major effect of nitrogen on Carmel hull samples with significant water treatment influence on low N samples. Error of classification on cross validation: high N high W 10%, high N low W 14%, low N low W 10%, low N high W 25%.

**Figure 7:**
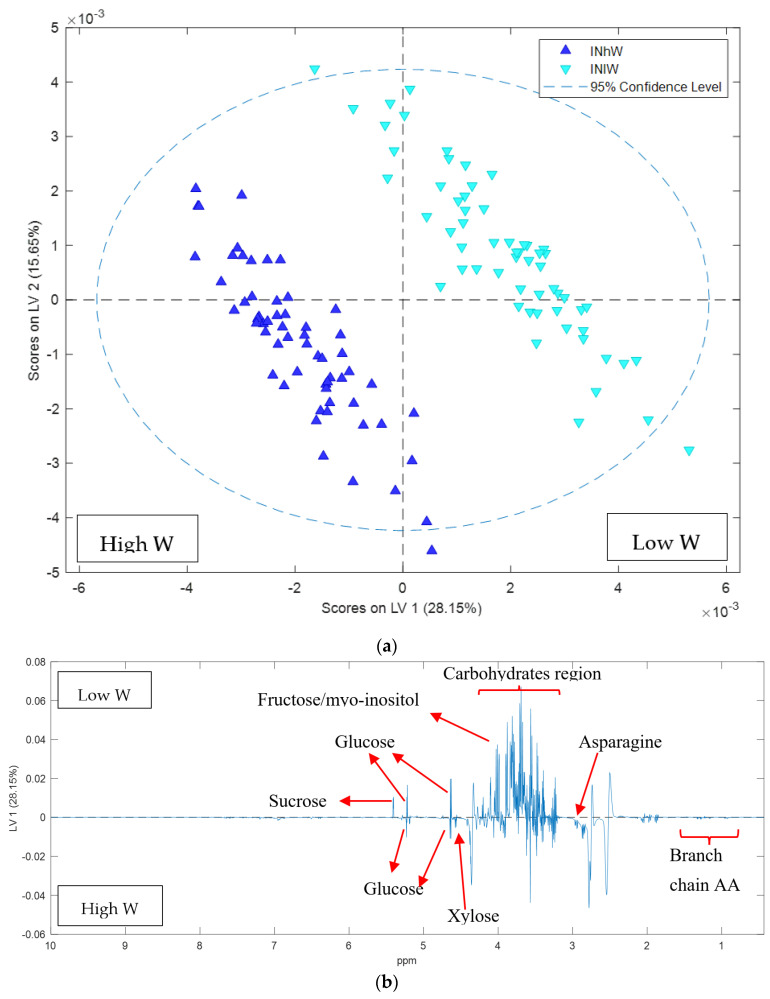

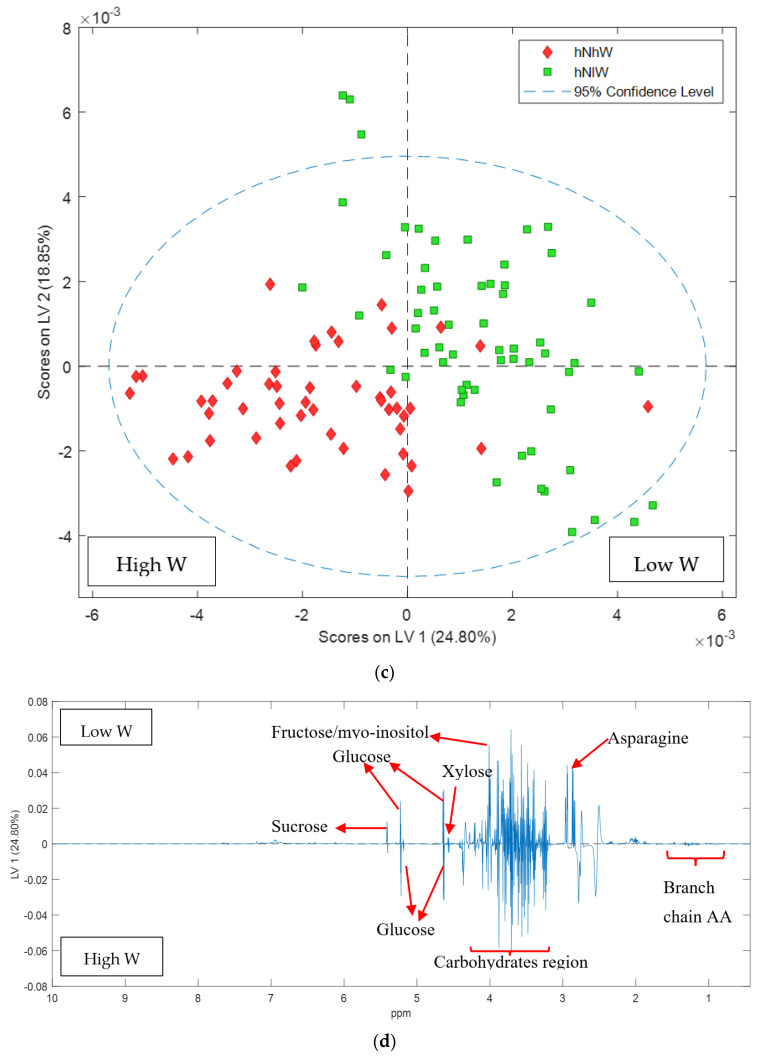
PLSDA score plot of ^1^H NMR data of Carmel hull samples: (**a**) PLSDA score plot showing effect of water treatments on hull composition of samples from low N treatment with classification error on cross validation of 3% and permutation *p*-value < 0.05 (**b**) Loadings plot of LV1 variable indicate the difference in hull composition components under water treatments of low N treatment (**c**) PLSDA score plot showing effect of the high N treatment on hull composition with classification error on cross validation of 1% and permutation *p*-value < 0.05 (**d**) Loadings plot of LV1 variable indicate the differences in hull composition components under water treatments with high N treatment.

To explore the effect of the water treatments on Carmel hull samples, classification modelling was conducted using PLS-DA to confirm the PCA results. An OSC processed PLS-DA model was conducted with classification based on the four treatment classes. The OSC processed PLS-DA model was predictive for each class with classification errors between 10% and 25% on cross validation: high N high W 10%, high N low W 14%, low N low W 10%, low N high W 25% (Figure 6). When the data was modelled according to high or low N the error on cross validation was 0, demonstrating the significance of the nitrogen treatment. There was a significant effect of the water treatment in both nitrogen treatments, particularly under low N (Figure 6). When the individual nitrogen treatments were analysed separately clustering due to water treatment was observed. The PLS-DA model of low N spectra showed a strong influence of water treatment with classification error on cross validation of 1% and permutation *p*-value < 0.05 indicating the model significance at the 95% confidence level (Figure 7a). The PLS-DA loadings plot analysis showed the sugars are elevated under the low W conditions while asparagine and branch chain amino acids are reduced (Figure 7b). The OSC processed PLS-DA model of high N samples also showed separation based on water treatment classification (Figure 7c). The classification error on cross validation samples was 3% with permutation *p*-value < 0.05. It was also observed that under high N low W, asparagine and sucrose were elevated (Figure 7d).

## 3. Discussion

Analysis of Nonpareil and Carmel almond varieties revealed that the two varieties are distinguishable from one another on the basis of their hull composition. Nonpareil hulls were higher in total sugar content and had more asparagine and branched chain amino acids than Carmel hulls. This study has also shown that the hull composition of both almond varieties changed when the restricted nitrogen and water treatments were applied to the trees. Nitrogen had the major influence on hull composition. Under high nitrogen, water had minimal influence in both Nonpareil and Carmel whereas under low nitrogen, water had a significant influence. The major changes occurred in concentrations of the sugars glucose and sucrose, and the amino acid asparagine. In the Nonpareil hulls, glucose is relatively elevated under high nitrogen and high water and low nitrogen and low water. For Carmel hulls the pattern of asparagine accumulation is the opposite, with asparagine relatively elevated under high nitrogen and low water and low nitrogen with high water (Table 1). These changes in the hull composition under different nitrogen and water scenarios have the potential to be affecting the growth of *R. stolonifer* and its metabolite production in hull rot disease.

There have been a few studies on the almond hull composition for animal feed nutrition [25,26,28]. Sequeira et al. determined the individual carbohydrate constituents of two ground almond hull samples from northern California using gas liquid chromatography [25]. They identified sucrose, fructose, glucose and two hexitols, sorbitol and inositol, in the almond hull samples. The results showed that total carbohydrate was 31.5% along with copper reducing water, moisture, water soluble solids, methanol soluble solids and ash [25]. Calixto et al. analysed mineral elements and protein amino acid makeup along with total nitrogen and sugar, moisture, ash, fat and crude fibre of almond hulls in Mallorca, Spain [26]. The quantitative determinations of amino acids in extracted protein were constructed using an automatic amino acid analyser, Liquimat III (Kontron, Saint-Quentin-en-Yvelines, France), that used a ninhydrin reaction technique [26]. The amino acid composition comprised both essential and non-essential amino acids, the most abundant of which were aspartic acid and glutamic acid. The quantitative analysis of sugars by high performance thin layer chromatography (HPTLC) detected sucrose, glucose and fructose, similar to Sequeria et al. Following this, Calixto et al. reported the water-soluble carbohydrate composition of various parts of the almond fruits such as hulls, shells, integument and kernels using gas chromatography [27]. They determined sucrose as the major component in all the parts of almond fruit. Hulls, shells and integuments contained significant amounts of glucose, fructose, inositol and sorbitol; however, kernels contained only traces of these components [27]. In 2020, DePeters et al. examined the nutritional composition of hulls of different almond varieties Nonpareil, Butte, Mission and Padre to better formulate diets for lactating dairy cows [28]. They reported that Nonpareil had greater sugar content and lower content of ash, lignin and neutral detergent fibre compared to the other varieties [28]. In our present study, we identified similar carbohydrate compositions, i.e., sucrose, glucose, fructose and xylose in the hulls of Australian-grown Nonpareil and Carmel. However, we also identified free amino acids including asparagine which has not been previously reported. The ratio of the sugars and amino acids changed with variety and according to the water/nitrogen treatments that the trees were subjected to in the orchard.

This is of interest, as nitrogen and water treatments provided to the almond trees are documented to influence the development of hull rot in Californian orchards [8,9,10,11,31]. Restricting water supply to trees before harvest or using regulated deficit irrigation significantly reduced hull rot [8,9,10]. Similarly, reducing the nitrogen supply reduced hull rot severity [11,12]. In our study, while the biggest differences were attributable to variety, with Nonpareil hulls higher in total sugars and asparagine than Carmel hulls, significant differences were also observed in the sugar and amino acid composition of both Carmel and Nonpareil hulls when grown under different nitrogen and water treatments. In Nonpareil under standard practice (high N high W), glucose and asparagine were elevated, but sucrose was not elevated. By restricting the water (high N low W), this was reversed, and sucrose was elevated, but glucose and asparagine were not elevated. When standard irrigation was applied but nitrogen was restricted (low N high W), glucose was elevated, but sucrose and asparagine were not elevated. Additionally, when both nitrogen and water were restricted (low N low W), asparagine was elevated, sucrose was increased, and glucose was reduced. In Carmel under standard practice (high N high W), glucose was elevated, but sucrose and asparagine were not. Restricting the water (high N low W) resulted in all three (sucrose, glucose and asparagine) being elevated. Under standard irrigation but restricted nitrogen (low N high W), glucose and asparagine were elevated, but sucrose was not. Restricting both nitrogen and water (low N low W) resulted in elevated sucrose, reduced glucose and asparagine. These observed changes in sugars and asparagine in response to restrictions in nitrogen and water applied in the orchard may help explain why the severity of hull rot is reduced under restricted water and nitrogen conditions. It is well documented that acid production by *Rhizopus stolonifer* and other *Rhizopus* species is influenced by the carbon and nitrogen sources available during their growth [14,21,32].

*Rhizopus* species, can produce large amounts of fermentation products such as ethanol, L(+)-lactic acid, fumaric acid and to a lesser extent malic acid that have commercial uses in the food and feed industry [13,14]. Organic acid production depends on various factors such as the carbon source utilised by *Rhizopus* spp., oxygen supply, nitrogen limitation and fungal strain selection [21,33]. Studies have found that several carbon sources (glucose, sucrose, mannose, xylose, fructose, cellobiose, fatty acids and glycerol) can be used as a substrate for lactic acid and fumaric acid production [13,15,21,33,34]. For example, Kenealy et al. demonstrated that *R. arrhizus* produced 97 g L^−1^ fumaric acid (9.7% *w*/*w*) when supplied with 120 g L^−1^ glucose as a carbon source [35]. Moon et al. observed the effect of various carbon sources at 50 g L^−1^ concentration on fumaric acid production by a *Rhizopus* sp. strain isolated from brown rice [36]. The carbon sources included in the study were glucose, sucrose, fructose, glycerol, lactose, maltose, starch and galactose. The results showed *Rhizopus* strain could metabolise glucose and fructose rapidly followed by maltose, starch and galactose, and lastly, glycerol. The highest concentration of fumaric acid 16 g L^−1^ was obtained with glucose as the carbon source. In contrast, no fumaric acid production was observed using sucrose as the carbon source. In our study, we also found similar carbohydrates, i.e., glucose, sucrose, fructose and xylose present in the almond hulls. These sugars were higher in Nonpareil than Carmel, and Nonpareil is known to be more susceptible to hull rot disease. In addition, Nonpareil hulls grown under high nitrogen and high-water conditions contained elevated levels of both glucose and asparagine which was not the case for Carmel hulls. These sugars are likely to be providing the substrate for *R. stolonifer* to produce fumaric acid in hull rot disease.

Along with specific carbohydrate sources, i.e., the sugars glucose, sucrose, fructose, xylose or a mixture of these sugars, *Rhizopus* species also require a nitrogen source to produce organic acids. In the bio-fermentation industries, inorganic nitrogen sources such as urea and ammonium sulphate ((NH_4_)_2_SO_4_) are used to enhance fumaric acid production by increasing metabolic activities of fungal cells over fungal growth [33]. *Rhizopus* can metabolise amino acids as nitrogen sources [37,38,39] but this can be economically challenging for large scale production of organic acids [40]. In our study, we found high concentrations of asparagine inside the hull in both almond varieties. Asparagine has not been reported in other hull composition studies and has not been included in *Rhizopus* growth studies. Therefore, this high concentration of asparagine could potentially provide a nitrogen source for *R. stolonifer* to produce fumaric acid inside a hull along with the carbohydrate sources, i.e., glucose, sucrose, fructose and xylose also present in the hull. The results from this study describing the presence of amino acids (especially relatively large amounts of asparagine) and sugars in almond hulls will allow informed design of *Rhizopus* metabolism studies to ascertain whether it utilises asparagine and carbohydrates, i.e., glucose, fructose, sucrose and xylose present in the almond hull to produce fumaric acid.

In summary, this current study has shown that when grown under Australian conditions, Nonpareil and Carmel almond hulls contain sugars such as glucose, sucrose, fructose and xylose and amino acids, in particular asparagine. The relative concentrations of sugars and amino acids change according to variety and the availability of nitrogen and water to the trees. *Rhizopus* fungi prefer simple sugars such as glucose and amino acids for their growth, producing organic acids as by-products. The higher concentration of sugars and asparagine in Nonpareil may explain why Nonpareil is more susceptible than Carmel to the disease hull rot, symptoms of which are caused by fumaric acid production by *Rhizopus stolonifer* growing within the hull. In general, restricting nitrogen and water to the trees reduced the asparagine and glucose content of the hulls, creating a less favourable environment for acid production, which may explain the reduction in hull rot.

## 4. Materials and Methods

### 4.1. Site Description

The experimental site was located on a commercial orchard at Lindsay Point, Victoria, planted in 2005 with Nonpareil as the main variety and Carmel as a pollinator. The orchard rows were oriented east/west with row and tree spacings of 7.2 × 4 m, respectively. The experimental layout was a replicated block design with four treatments randomised within six blocks, giving six replicates per treatment. Each replicate plot was three rows wide and 10 trees long. The experimental unit was the central four trees per variety within the replicate plot.

The treatments consisted of two levels of irrigation (high and low) and two levels of nitrogen (high and low). High N was the commercial practice recommended by the Almond Board of Australia (ABA) for the major mineral elements N:P:K 320:40:400. The low N treatment was applied as 56% of the high N (N:P:K 180:40:400). The high-water treatment was irrigation at 100% ETc estimated using the ABA-recommended crop factors. The low water treatment was applied as 70% ETc throughout the growing season as a sustained deficit. The irrigation and nitrogen treatments were established four years prior to the start of the experiment, and the experiment was conducted during the 2019–2020 season.

### 4.2. Sample Collection

Almond fruit of Nonpareil and Carmel varieties were collected in January 2019 at the early stage of hull split (less than 1 cm suture), which is the stage most susceptible to *Rhizopus stolonifer* infection. Twenty healthy fruit were selected from the four trees per replicate. Collected fruits were separated into hull, shell and kernel. All the samples were snap frozen in liquid nitrogen and stored in paper bags at −80 °C for at least 24 h until they could be further processed. After that, samples were freeze-dried in an ALPHA 1-4 LD freeze fryer (Martin Christ, Germany) at −53 °C and 0.030 m Bar pressure for 48 h. Freeze-dried samples were stored at 4 °C.

### 4.3. Sample Preparation for NMR

The freeze-dried hull samples were processed further for hull composition analysis. Ten hull samples per replicate from six replicates per treatment were used, resulting in 60 samples per treatment per variety. Individual freeze-dried hulls were transferred into 15 mL polycarbonate vials with 7/16” and 3 mm stainless steel grinding balls. Sample vials were placed on a Geno/Grinder 2010 (SPEX Sample Prep, Metuchen, NJ, USA) and hulls were homogenised at 1200 rpm for 2 min. The resultant fine powder was stored at 4 °C until ready to be weighed, then 50 mg ± 0.02 mg of powder from each sample added to 2 mL Eppendorf tubes.

Deuterium oxide (D_2_O, Cambridge Isotope Laboratories, Massachusetts) solution was prepared twice with the addition of 5 mM 4,4-Dimethyl-4-silapentane-1-sulfonic acid (DSS-d_6_, Sigma-Aldrich, St. Louis, MO, USA) deuterated solvent and other with not deuterated solvent for Nonpareil and Carmel samples sets, respectively; the solution had a pH of 6.9. Hull samples were extracted with 1000 µL of deuterium oxide (D_2_O) solution. Subsequently, samples were subjected to 30 sec vortex (Ratek vortex mixer, VM1, Boronia, VIC, Australia) and 5 min sonication (SoniClean, 250TD, Stepney, SA, Australia) followed by 15 min centrifugation (16,100× *g*, 21 °C). An aliquot of 550 µL was taken from the supernatant layer and transferred into 5 mm NMR tubes.

### 4.4. ^1^H NMR Spectroscopy Analysis of the Almond Hull Composition

Standard 1D NMR spectra were obtained on a Bruker 700 MHz Avance^TM^ III NMR instrument equipped with a cryoprobe and a SampleJet automatic sample changer with cooling (Bruker Biospin, Rheinstetten, Germany). All 1D NMR spectra were carried out using Bruker noesypr1d pulse sequence over −4 to 14 ppm spectral range by suppression of water resonance by pre-saturation. Acquisition parameters were as follows: spectral width, 11.08 ppm; acquisition time, 2.11 s per scan; time domain points, 32 K; and number of scans, 128 and 8 dummy scans. A line broadening of 0.3 Hz was applied to all spectra prior to Fourier Transformation. A total of 472 spectra were manually phased and baseline corrected in Topspin 4.0 (Bruker Biospin, Rheinstetten, Germany) and referenced to the DSS-d_6_ or DSS not deuterated at 0.0 ppm. Assigned peaks were identified using Chenomx NMR suit software v.8.6 (Chenomx Inc., Edmonton, AB, Canada).

### 4.5. Data Processing and Multivariate Statistical Data Analysis

All spectra were imported into Matlab (R2018a, Mathworks, Natick, MA, USA) using ProMetab_v1_1 script for data pre-processing. The residual DSS peak was manually removed (4.888–4.808 ppm) before data were further assessed. Statistical analysis of NMR data was carried out using PLS toolbox (Ver 8.6.1, Eigenvector Research, Wenatchee, WA, USA). Data was pre-processed prior to Principal Component Analysis (PCA) or Partial Least Squares Discriminant Analysis (PLS-DA). Pre-processing includes normalisation (total spectral area normalised to 1), baseline adjustment (automatic weighted least squares, order = 2) and mean centering.

In the PLS-DA analysis, samples were classified into the separate group or classes known to the model. The y-block (class) was auto-scaled for PLS-DA and cross validation was based on the random subset with ten data splits and five iterations on the entire NMR data set. The orthogonal signal correction (OSC) was used as pre-processing to improve the classification and visualisation of the PLS-DA model. Statistical significance of obtained PLS-DA model was evaluated with permutation testing.

## Figures and Tables

**Table 1:** Treatment effects causing relative increase in sucrose, glucose and/or asparagine in hulls of Nonpareil and Carmel almonds (from Figure 4 and Figure 7).

Treatments	Nonpareil	Carmel
High N High W	Glucose	
	Asparagine	
High N Low W	Sucrose	Sucrose Asparagine
Low N High W	Glucose	Asparagine
Low N Low W	Sucrose	Sucrose Glucose
	Asparagine	

## Data Availability

The data presented in this study are available on request from the corresponding author.

## Supplementary Materials

The following are available online at https://www.mdpi.com/article/10.3390/metabo11100674/s1, Figure S1. Comparison of Nonpareil and Carmel hull components based on loadings plot, Figure S2. PCA score and loadings plots of ^1^H NMR data of Nonpareil hull, Figure S3. PCA score and loadings plots of ^1^H NMR data of Carmel hull, Table S1. Components have been identified in the almond hull of Nonpareil and Carmel varieties.

## References

[1] Teviotdale B.L., Michailides T.J., Pscheidt J.W. (2002). Compendium Of Nut Crop Diseases In Temperate Zones.

[2] Gouk C. (2016). Almond diseases and disorders. Acta Hortic. Int. Soc. Hortic. Sci..

[3] Cline H. (2008). Hull rot is the “gout” of almond diseases. West. Farm Press.

[4] Mirocha C.J., Wilson E.E. (1961). Hull rot disease of almonds. Phytopathology.

[5] Adaskaveg J.E. Epidemiology, and control of fungal and bacterial diseases of almond. Proceedings of the Almond Conference: Disease and Aflatoxin Management Update.

[6] Kreidl S., Wiechel T., Faulker P., Tesoriero L., Edwards J. (2021). Hull rot of almond. Nutshell—Autumn.

[7] Mirocha C.J., DeVay J.E., Wilson E.E. (1961). Role of fumaric acid in the hull rot disease of almond. Phytopathology.

[8] Teviotdale B.L., Michailides T.J., Goldhamer D.A., Viveros M., Schmidt L., Hines V. (1994). Cutting off irrigation early may reduce almond hull rot. Calif. Agric..

[9] Teviotdale B.L., Michailides T.J., Goldhamer D.A., Viveros M. (1995). Reduction of almond hull rot disease caused by *Rhizopus stolonifer* by early termination of preharvest irrigation. Plant Dis..

[10] Teviotdale B.L., Goldhamer D.A., Viveros M. (2001). Effects of deficit irrigation on hull rot disease of almond trees caused by *Monilinia fructicola* and *Rhizopus stolonifer*. Plant Dis..

[11] Teviotdale B.L. (1994). Effect of Four Level of Applied Nitrogen on Three Fungal Diseases of Almond Trees. Proceedings of the 22nd Almond Research Conference.

[12] Saa S., Peach-Fine E., Brown P., Michailides T., Castro S., Bostock R., Laca E. (2016). Nitrogen increases hull rot and interferes with the hull split phenology in almond (*Prunus dulcis*). Sci. Hortic..

[13] Magnuson J.K., Lasure L.L., Tkacz J.S., Lange L. (2004). Organic acid production by filamentous fungi. Advances in Fungal Biotechnology for Industry, Agriculture, and Medicine.

[14] Goldberg I., Rokem J.S., Pines O. (2006). Organic acids: Old metabolites, new themes. J. Chem. Technol. Biotechnol..

[15] Ward G.E., Lockwood L.B., May O.E., Herrick H.T. (1936). Biochemical studies in the genus *Rhizopus*. I. The production of dextro-lactic acid. J. Am. Chem. Soc..

[16] Foster J.W., Waksman S.A. (1939). The production of fumaric acid by molds belonging to the genus *Rhizopus*. J. Am. Chem. Soc..

[17] Maas R.H.W., Bakker R.R., Eggink G., Weusthuis R.A. (2006). Lactic acid production from xylose by the fungus *Rhizopus oryzae*. Appl. Microbiol. Biotechnol..

[18] Zhang Z.Y., Jin B., Kelly J.M. (2007). Production of lactic acid from renewable materials by *Rhizopus* fungi. Biochem. Eng. J..

[19] Nwokoro O. (2015). Studies on the production of citric acid by *Rhizopus stolonifer*. Am. Chem. Sci. J..

[20] Roa Engel C., Straathof A., Zijlmans T., Gulik W., Wielen L. (2008). Fumaric acid production by fermentation. Appl. Microbiol. Biotechnol..

[21] Lennartsson P.R., Taherzadeh M.J., Edebo L., Batt C.A., Tortorello M.L. (2014). Rhizopus. Encyclopedia of Food Microbiology.

[22] Kapilan R. (2015). Enzyme production by *Rhizophus stolonifer* isolated from bread and kinetic properties of the extracellular amylase. Jacobs J. Enzymol. Enzym. Eng..

[23] Papadaki A., Androutsopoulos N., Patsalou M., Koutinas M., Kopsahelis N., Machado de Castro A., Papanikolaou S., Koutinas A. (2017). Biotechnological production of fumaric acid: The effect of morphology of *Rhizopus arrhizus* NRRL 2582. Fermentation.

[24] Martin-Dominguez V., Estevez J., Ojembarrena D.F., Santos E.V., Ladero M. (2018). Fumaric acid production: A biorefinery perspective. Fermentation.

[25] Sequeira R.M., Lew R.B. (1970). Carbohydrate composition of almond hulls. J. Agric. Food Chem..

[26] Calixto F.S., Cañellas J. (1982). Chemical composition of hulls of the sweet almond (*Prunus amygdalus*). J. Sci. Food Agric..

[27] Calixto F.S., Canellas J., Garcia-Raso A. (1984). Gas chromatographic analysis of sugars and sugar-alcohols in the mesocarp, endocarp, and kernel of almond fruit. J. Agric. Food Chem..

[28] DePeters E.J., Swanson K.L., Bill H.M., Asmus J., Heguy J.M. (2020). Nutritional composition of almond hulls. Appl. Anim. Sci..

[29] Aguilar A.A., Smith N.E., Baldwin R.L. (1984). Nutritional value of almond hulls for dairy cows. J. Dairy Sci..

[30] Abreu A.C., Fernández I. (2020). NMR metabolomics applied on the discrimination of variables influencing tomato (*Solanum lycopersicum*). Molecules.

[31] Goldhamer D.A., Viveros M. (2000). Effects of preharvest irrigation cutoff durations and postharvest water deprivation on almond tree performance. Irrig. Sci..

[32] Meussen B., Graaff L., Sanders J., Weusthuis R. (2012). Metabolic engineering of *Rhizopus oryzae* for the production of platform chemicals. Appl. Microbiol. Biotechnol..

[33] Das R.K., Brar S.K., Verma M., Kaur Brar S., Jyoti Sarma S., Pakshirajan K. (2016). Fumaric Acid: Production and Application Aspects. Platform Chemical Biorefinery.

[34] Kowalczyk S., Komoń-Janczara E., Glibowska A., Kuzdraliński A., Czernecki T., Targoński Z. (2018). A co-utilization strategy to consume glycerol and monosaccharides by *Rhizopus* strains for fumaric acid production. AMB Express.

[35] Kenealy W., Zaady E., du Preez J.C., Stieglitz B., Goldberg I. (1986). Biochemical aspects of fumaric acid accumulation by *Rhizopus arrihzus*. Appl. Environ. Microbiol..

[36] Moon S.K., Wee Y.J., Yun J.S., Ryu H.W. (2004). Production of fumaric acid using rice bran and subsequent conversion to succinic acid through a two-step process. Appl. Biochem. Biotechnol..

[37] Stocks P.K., Ward B.Q. (1962). Utilization of amino acids by *Rhizopus nigricans*. Can. J. Microbiol..

[38] Sorenson W.G., Hesseltine C.W. (1966). Carbon and nitrogen utilization by *Rhizopus oligosporus*. Mycologia.

[39] Thanh N.V., Rombouts F.M., Nout M.J.R. (2005). Effect of individual amino acids and glucose on activation and germination of *Rhizopus oligosporus* sporangiospores in tempe starter. J. Appl. Microbiol..

[40] Clarke K.G. (2013). Microbial kinetics during batch, countinuous and fed-batch processes. Bioprocess Engineering: An Introductory Engineering and Life Science Approach.

