# Supplementary material for: Restricted Nitrogen and Water Applications in the Orchard Modify the Carbohydrate and Amino Acid Composition of Nonpareil and Carmel Almond Hulls
Source: Metabolites. 2021 Sep 30;11(10):674. doi: 10.3390/metabo11100674 (PMC8538458; doi:10.3390/metabo11100674)
Supplement: Supplementary file 1 [file metabolites-11-00674-s001.zip › metabolites-1358827- supplementary final.pdf]

metabolites

Supplementary Data

Restricted nitrogen and water applications in the orchard modify the carbohydrate and amino acid composition of Nonpareil and Carmel almond hulls

Anjali Zaveri [1*], Simone Rochfort [1,2] and Jacqueline Edwards [1,2]

[1] School of Applied Systems Biology, La Trobe University, Bundoora, VIC 3083, Australia
[2] Agriculture Research Victoria, 5 Ring Road, Bundoora, VIC 3083, Australia
* Correspondence: anjali.zaveri@agriculture.vic.gov.au;

Table S1. Components have been identified in the almond hull of Nonpareil and Carmel varieties.

Components identified	Nonpareil	Carmel
Glucose	✓	✓
Sucrose	✓	✓
Fructose	✓	✓
Xylose	✓	✓
Asparagine	✓	✓
Alanine	✓	✓
Threonine	✓	✓
Valine	✓	✓
Isoleucine	✓	✓
Leucine	✓	✓

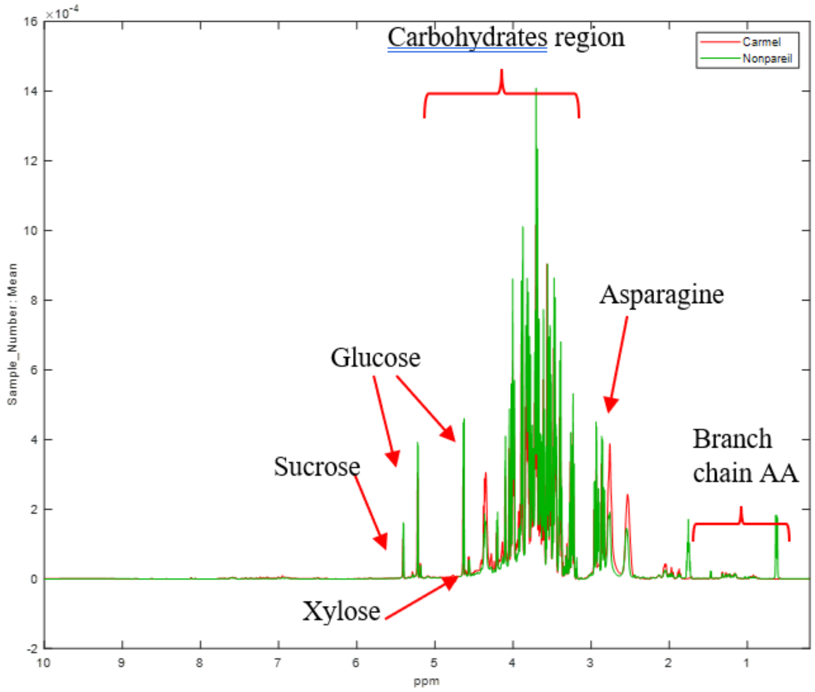

(a)

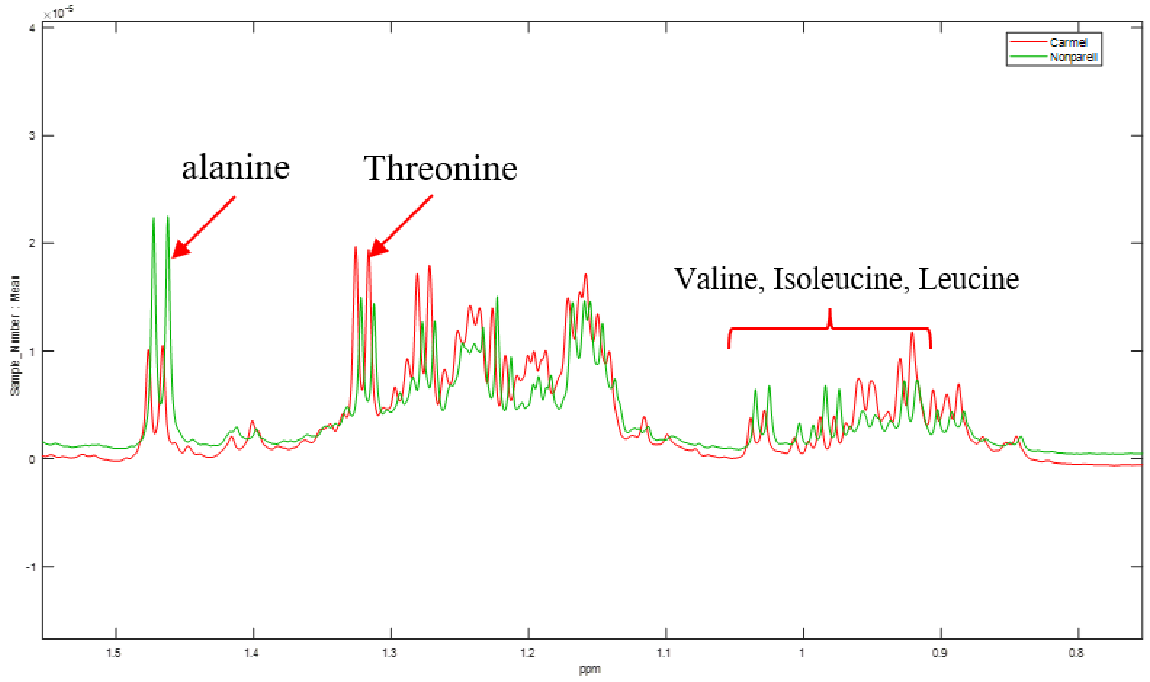
×10^-5
Carmel
Nonpareil
alanine
Threonine
Valine, Isoleucine, Leucine
Sample_Number : Mean
4
3
2
1
0
-1
1.5
1.4
1.3
1.2
1.1
1
0.9
0.8
ppm

(b)

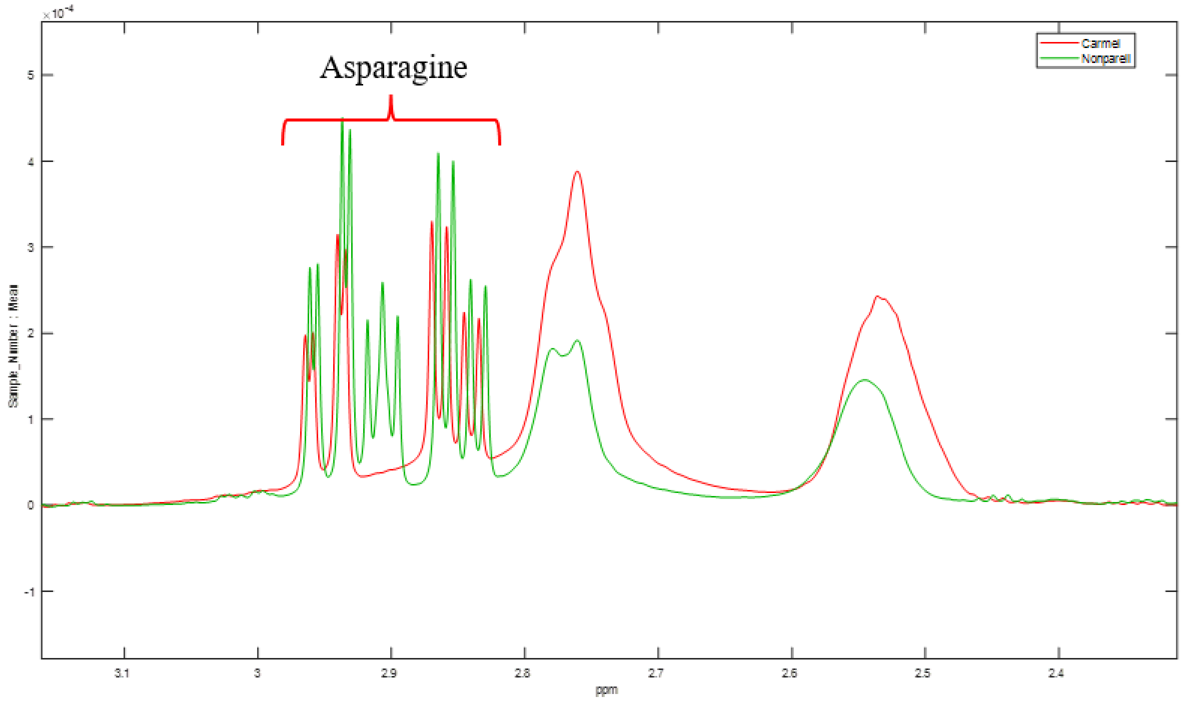
×10^-4
Carmel
Nonpareil
Asparagine
Sample_Number : Mean
5
4
3
2
1
0
-1
3.1
3
2.9
2.8
2.7
2.6
2.5
2.4
ppm

(c)

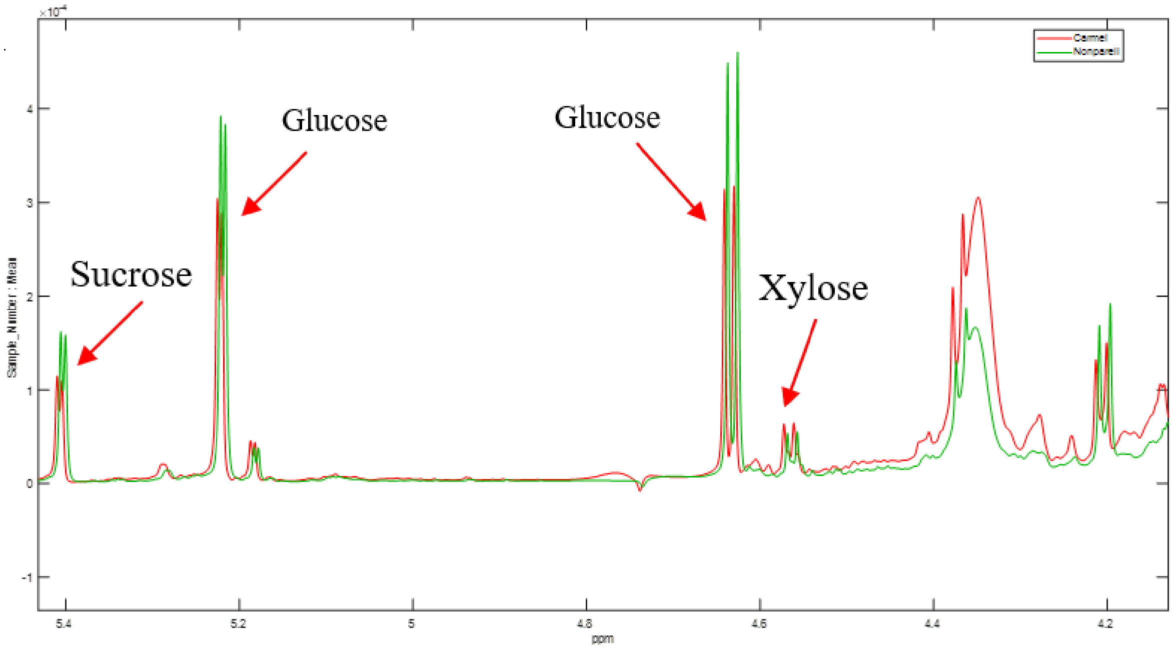

(d)

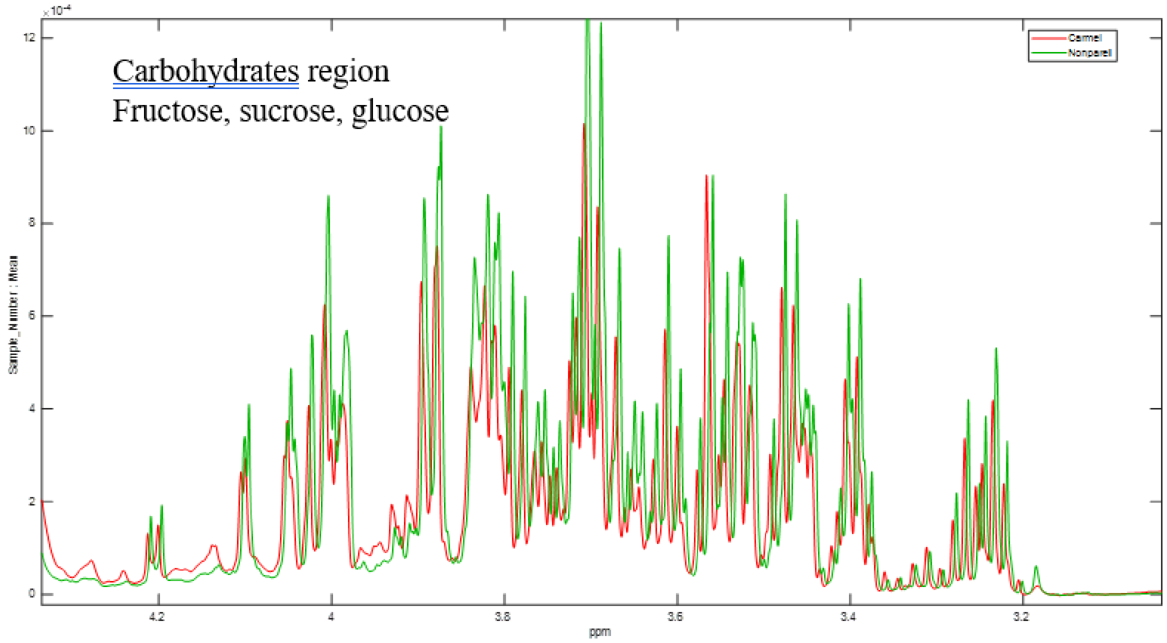

(e)

Figure S1. Comparison of Nonpareil and Carmel hull components based on loadings plot: components of interest have been identified in almond hull composition of both varieties - glucose, sucrose, fructose, xylose and branch chain amino acids (**a**) overlap of Nonpareil and Carmel spectra (**b**) amino acid region: Carmel has elevated level of amino acids (**c**) Elevated asparagine presence in Nonpareil samples (**d**) Elevated Glucose (4.51ppm, 5.2ppm), and Sucrose (5.4 ppm) (**e**) overlapping carbohydrate region: elevated level of carbohydrates in Nonpareil samples.

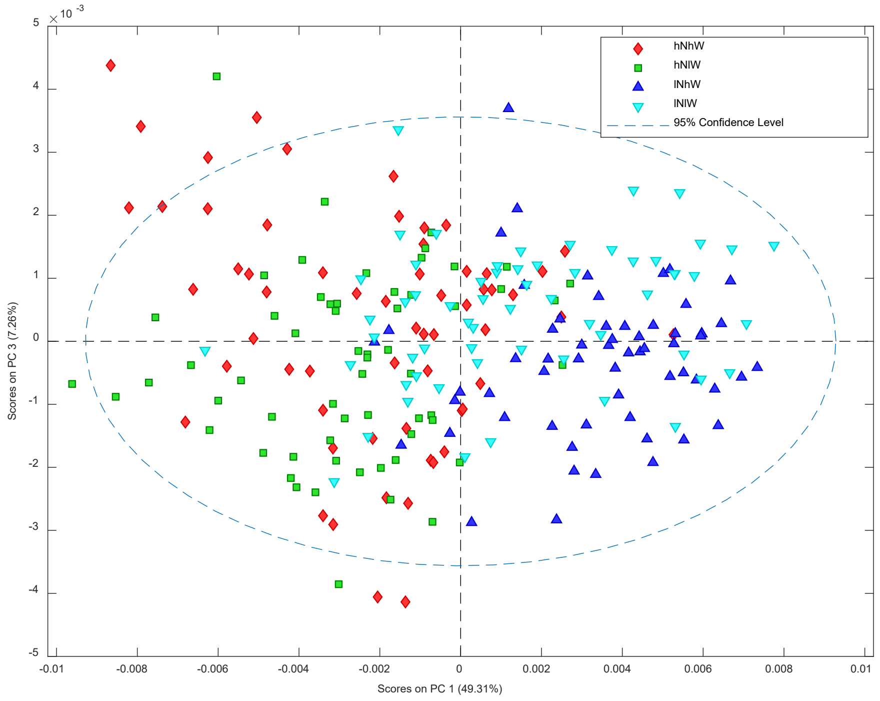

(**a**)

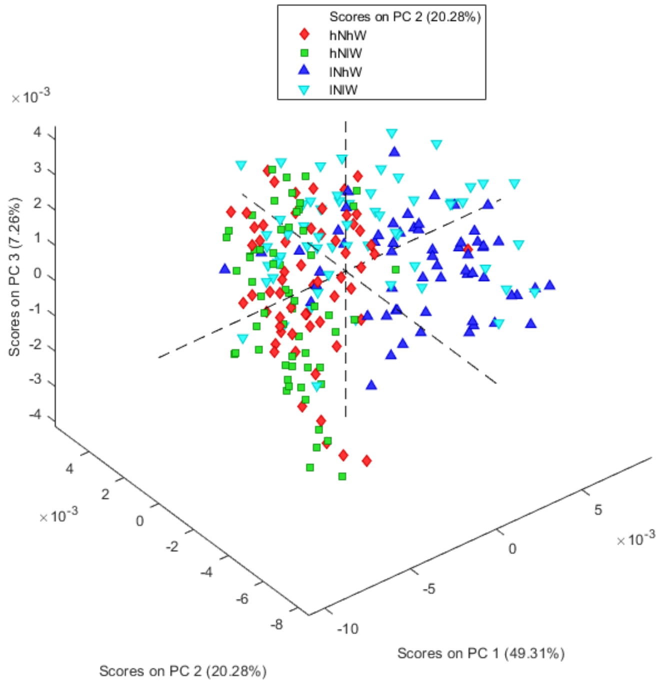

(**b**)

Figure S2. PCA score and loadings plots of ^{1}H NMR data of Nonpareil hull: (**a**) PCA scores plot showing separation of Nonpareil hull samples based on nitrogen treatment; (**b**) PCA 3D scores plot showing separation of hull samples based on nitrogen treatment.

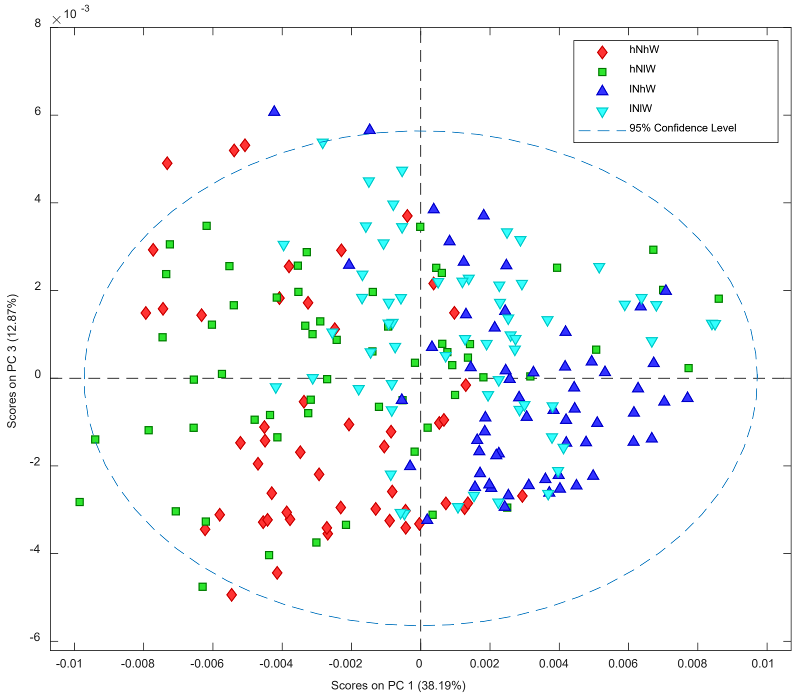

(**a**)

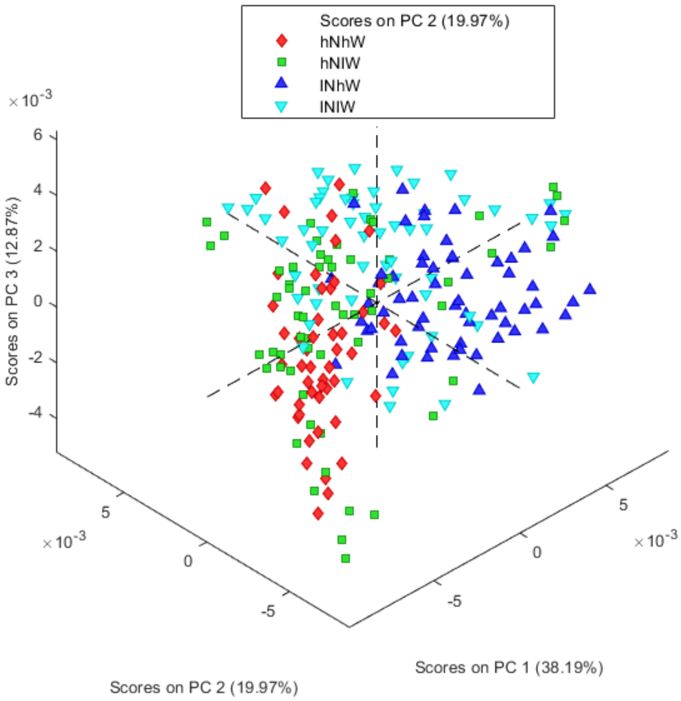

(**b**)

Figure S3. PCA score and loadings plots of ^{1}H NMR data of Carmel hull: (**a**) PCA scores plot showing separation of Carmel hull samples based on nitrogen treatment; (**b**) PCA 3D scores plot showing separation of hull samples based on nitrogen treatment.
